# Urotherapist activities in caring for patients with pelvic floor disorders: a prospective single-center observational study

**DOI:** 10.1007/s00404-020-05810-0

**Published:** 2020-09-30

**Authors:** Verena Geissbuehler, Susanne Forst, Matthias Werner, Cora-Ann Schoenenberger, Ruth Berner, Cornelia Betschart

**Affiliations:** 1 Department of Obstetrics and Gynecology, Cantonal Hospital Winterthur, Brauerstrasse 15, 8401 Winterthur, Switzerland; 2 Clinic of Gynecology/Gynecologic Oncology, St. Claraspital Basel, Basel, Switzerland; 3 Department of Gynecology, University Hospital Zurich, Zurich, Switzerland

**Keywords:** Advanced nurse practitioner, Urotherapy, Pelvic floor disorders, Patient satisfaction, Interprofessional collaboration

## Abstract

**Purpose:**

Patients with pelvic floor disorders are growing in number. The aim of this study is to outline the main activities of a urotherapist, an advanced nurse practitioner, in the care of patients with pelvic floor disorders and to evaluate patient satisfaction with the service urotherapists provide.

**Methods:**

The prospective single-center observational study was carried out from July 2016 to June 2018. Parameters used to assess the urotherapist activities included the number of consultations, type of counselling, time frame of consultations and therapy and patient satisfaction. In a subgroup of 38 patients, satisfaction with the urotherapy sessions was evaluated by a questionnaire.

**Results:**

Totally, 1709 patients were examined by urogynecologists. Five hundred and fourteen (30%) with chronic pelvic floor disorders were subsequently referred to a urotherapist. Of these patients, 60% were at least 65 years old. The most common pelvic floor disorders (221 patients; 43%) were an overactive bladder, recurrent urinary tract infections, chronic cystitis and pelvic pain syndrome; the second most common pelvic floor disorder was pelvic organ prolapsed (151 patients; 29%). Of the study subgroup comprising 38 patients, 32 (84%) returned the patient satisfaction questionnaire. All 32 patients specified their level of agreement with the urotherapist’s professional competence, empathy, temporal availability and quality of advice as “agree to strongly agree.”

**Conclusions:**

Management by a urotherapist was highly appreciated. The role of the urotherapist as a care coordinator, their level of autonomy and barriers to the implementation in primary care requires further exploration.

**Electronic supplementary material:**

The online version of this article (10.1007/s00404-020-05810-0) contains supplementary material, which is available to authorized users.

## Introduction

Pelvic floor disorders (PFDs) consisting of an overactive bladder, chronic bladder diseases and urinary stress incontinence, recurrent urinary tract infections, pelvic organ prolapse and fecal incontinence [1, 2] are often chronic diseases that may require regular assistance over long periods of time [3]. The present worldwide increase in the proportion of older people (aged 65 years and over) is accompanied by a rise of pelvic floor disorders and multimorbidity [3, 4]. Pelvic floor disorders considerably reduce the well-being and quality of life of the affected by interfering with daily and social activities, which in the worst case leads to isolation [5, 6].

To overcome the health care needs of an aging population with a variety of chronic diseases, new treatment and management concepts, such as interprofessional collaborations, are emerging and have already been implemented in different settings [7–9]. In 2010, the World Health Organization (WHO) [10] launched a call for collaboration concepts between different medical professional groups, especially between nurses and doctors. A care coordinator for people with multimorbidity seems to be pivotal for high-quality clinical work, the likelihood to continue living in the community and improved self-management by patients with chronic diseases. Furthermore, such integrated care programs aim to stabilize health care costs [11]*.*

Interprofessional collaborations represent a new strategy designed to cover increasing patient demands with respect to medical, psychological and social skills and consultation time. As a consequence, the curricula of nurses and physicians will change, and the interactions/partnership between health care providers will increase accordingly. In particular, chronic conditions require the comprehensive involvement of medical, social and cultural aspects in an interprofessionally organized health care system that so far remains insufficiently explored [7, 9, 12].

The interprofessional approach for the treatment of pelvic floor disorders was initially introduced by pediatricians [13] whose clinical activities in treating children with urinary and fecal incontinence were supported by urotherapists, i.e., specialist nurses/advanced nurse practitioners [14, 15]. A review addressing urotherapy in children revealed that the urotherapist contributed to a reduced urinary and fecal incontinence [14]. However, information regarding the frequency, number and time of urotherapeutic consultations was not provided. Furthermore, comprehensive data on the clinical activities of urotherapists in adult urogynecology and their impact on the patient's quality of life are entirely lacking. In 2009, a nurse-led urogynecology triage clinic was established, offering a service for women with long-term urinary incontinence [16]. A retrospective study assessing the clinic led to the conclusion that patients with urinary incontinence were adequately managed by advanced nurse practitioners [17].

Growing interest in the work of advanced nurse practitioners motivated us to specifically examine the professional activities of the urotherapist. Like physicians in long-term care facilities and primary care for the aging population, urogynecologists do not have the capacity to meet all the needs of patients with pelvic floor disorders, especially when the complaints are chronic. In their review, Lovink et al. [18] concluded that the substitution of doctors by nurses achieves care processes and patient outcomes that are at least as good as those attained by doctors alone.

The aim of this study is to describe the main activities of the urotherapist in the care of patients with pelvic floor disorders and to evaluate patient satisfaction with the service urotherapists provide.

## Material and methods

The study was approved by the Ethic Committee of the Canton Zurich (Swissethics: BASEC 2016-00211) and performed from July 2016 to June 2018 in the outpatient clinic of the Department of Obstetrics and Gynecology, of the Cantonal Hospital Winterthur/Switzerland, a tertiary referral center. All participants provided written consent. The urotherapist documented the type of pelvic floor disorder, the type of counselling, the time frame of the treatments and the patient satisfaction with the service.

### Participants

Patients were included after a first consultation by a urogynecologist, if the decision made together with the urotherapist was that advanced care for pelvic floor disorders was required due to a probable complexity and chronicity of the urogynecological disease. Patients were grouped according to the following types of PFD: (1) overactive bladder with or without incontinence/recurrent urinary tract infections/chronic cystitis/pelvic pain syndrome (collectively termed increased bladder sensation complaints) [19], (2) pelvic organ prolapse, (3) stress urinary incontinence and (4) fecal incontinence. The patients were ≥ 16 years old and signed an informed consent for pseudonymized use of their medical records. Patients with comprehension difficulties (language) were excluded.

The percentage of patients undergoing subsequent urogynecological surgery in the groups with and without urotherapist counselling was documented.

A subanalysis was performed for a subgroup of patients that consisted of patients who were willing to participate in the prospective observational study by filling out a questionnaire.

The consultation fees were covered by general, mandatory health insurances.

### Data collection

Demographic and clinical data were collected for all patients. The clinical data were documented by the urotherapist and urogynecologist in an electronic medical records system (CGM Phoenix) and using the *R*esources *A*genda *P*lanning (RAP) (Polypoint): interprofessional planning and administration tool.

Consultation schedules, including the frequency and duration of individual visits, were organized according to patient needs. The number of consultations was not restricted. Management of PFDs ended when symptoms were resolved, when the patient was satisfied with the counselling or when the patient did not want any further intervention. Usually, there was a final consultation with the urotherapist or a urogynecologist. The referring doctors received a final report.

For the subgroup analysis, the German Pelvic Floor Questionnaire [20] was distributed by the urogynecologist or urotherapist at the first visit. The German Pelvic Floor Questionnaire consists of 42 questions related to bladder function, bowel function, prolapse symptoms and sexual function. The maximum total PFD score is 40 (greatest impairment). If a woman is not sexually active, the maximum score is 30 [20].

For the subgroup, detailed information regarding total consultation time, time in minutes per consultation and mode of consultation treatment was recorded on the study-specific case reporting form.

To evaluate patient satisfaction with the urotherapist and urogynecologist, a short, customized questionnaire assessing patient satisfaction, including a 5-point Likert scale (see Electronic supplementary material), was distributed at the last patient visit. The questionnaires were returned in person or by post (prepaid envelope provided).

### Training and activities of the urotherapist

Training as a general nurse is a prerequisite for advanced education as a urotherapist. The urotherapist training lasted one and a half additional years and included five modules focusing on diagnosis, treatment and care of people with functional, organic and neurogenic bladder disorders, and of patients with fecal incontinence. The training was completed by a written thesis and an oral exam.

The main activities of the urotherapist included instructions for lifestyle modifications, such as bladder training, bowel habit training, dietary changes, completion of drinking and micturition protocols, fitting of pessaries, teaching intermittent self-catheterization, changing indwelling urinary catheters and performing percutaneous posterior tibial nerve stimulation (PTNS) and bladder instillations. The urotherapist performed patient and nurse counselling in the outpatient clinic, communicated with nursing homes or conducted phone counselling.

### Statistics

Descriptive statistical values are expressed as means together with the standard deviation (SD) for parametric data, or as medians together with the range (minimum – maximum) for nonparametric data (e.g., age and consultation time). Absolute frequencies and percentages were calculated for nominal data (e.g., diagnosis of PFDs and activities of the urotherapist). *P*-values were calculated by the *t* test for independent samples, and a *p* value < 0.05 is considered as significant. Data were recorded and calculated using the IBM SPSS software (Version 22.0 for Windows; Chicago, Illinois/USA).

## Results

From July 2016 to June 2018, 1709 patients were examined by urogynecologists. Of these patients, 514 (30%) were subsequently referred to the urotherapist for different reasons, including the need for counselling for a chronic disease, fitting pessaries, conservative treatments, multimorbidity, age, etc.

### Main collective

Patient demographics, diagnosis and consultation characteristics are listed in Table 1.

**Table 1:** Patients comanaged by a urotherapist

	Main collective *n*=514	Subgroup *n*=38	Statistics
Average age (±SD), years	66.0 (±16.6)	71.6 (±14.0)	*p* = 0.038
< 65	204 (40%)	9 (24%)	
≥ 65	310 (60%)	29 (76%)	
Number of PFDs diagnosed			*p* = 0.000
Total	583	58	
Number per patient (±SD)	1.1( ± 0.4)	1.5 (±0.7)	
Pelvic floor disorders per patient			–
BSC	221 (43%)	33(87%)	
POP	151 (29%)	9 (24%)	
SUI	93 (18%)	10 (26%)	
FI	18 (4%)	6 (16%)	
Mixed disorders	100 (19%)	0	
Questionnaires			–
German pelvic floor questionnaire/dysfunction score, total (±SD)	na	8.41 (±4.63)	
Bladder domain		3.89 (±1.87)	
Bowel domain		2.66 (±1.61)	
Prolapse domain		1.01 (±1.88)	
Sexual function domain		0.73 (±1.61)	
Urotherapist consultations			–
Total	1555	330	
< 5 per patient	431 (84%)	17 (45%)	
≥ 5 per patient	83 (16%)	21 (55%)	
Type of consultation			–
Phone counselling	919 (59%)	88 (27%)	
Personal counselling	252 (16%)	40 (12%)	
Therapies (PTNS, pessaries)	18 (32%)	13 (72%)	
Catheter management	88 (6%)	56 (17%)	
Administrative support	113 (7%)	9 (3%)	
Consultation time, h (range) per patient			–
Urotherapist	1.3 (0.2–24.4)	57 (0.5–35.8)	
Urogynecologist	1.7 (0.3–12.5)	2.6 (0.5–10.8)	
Patients aged ≥65 and ≥5 consultations	62	17	–
BSC	45 (58%)	15 (65%)	
POP	17 (22%)	3 (17%)	
SUI	9 (13%)	2 (13%)	
FI	2 (4%)	1 (4%)	
Mixed disorders	2 (3%)	na	
Subsequent surgery	132 (26%)	7 (18%)	–

Patient demographics and diagnosis with mean, SD and range where appropriate Consultation characteristics

Percentages are given in brackets. Pelvic floor disorders (PFDs): *BSC* Summarized as increased Bladder Sensation Complaints, *POP* Pelvic Organ Prolapse, *SUI* Stress Urinary Incontinence, *FI* Fecal Incontinence and Mixed Disorders, *PTNS* percutaneous posterior tibial nerve stimulation

The 514 patients referred to the urotherapist had 583 PFDs in all, which correspond to 1.1 $\pm$ 0.4 PFD per patient on average; 221 patients (43%) suffered from an overactive bladder with or without incontinence/recurrent urinary tract infections/chronic cystitis/pelvic pain syndrome (summarized as increased bladder sensation complaints), 151 (29%) had pelvic organ prolapse, 93 (18%) suffered from stress urinary incontinence, and 18 (4%) had fecal incontinence. In all, 100 (17%) of the women did not only have PFDs but also additional urogynecological disorders (mixed disorders), such as vaginal adhesions after radiotherapy, vulvar diseases and menopausal disorders with negative impact on bladder function.

We distinguished two age groups: patients under 65 years (< 65) and patients 65 years and older (≥ 65), consisting of 204 (40%) and 310 (60%) patients, respectively.

The total number of urotherapeutic consultations received by the 514 patients was 1555; 431 patients (84%) had less than 5 consultations and 83 (16%) 5 or more consultations. The types of consultation were as follows: 919 phone counsellings, 252 personal counsellings, 183 therapies (such as percutaneous posterior tibial nerve stimulation (PTNS) or pessary fitting), 88 catheter management appointments and 113 administrative support sessions.

In all 62 of the 83 patients who had ≥ 5 consultations were ≥ 65. From these, 45 (58%) had an overactive bladder with or without incontinence/recurrent urinary tract infections/chronic cystitis/pelvic pain syndrome (summarized as increased bladder sensation complaints).

On average, each patient in the main collective received 1.3 h (0.2–24.4 h) of urotherapist consultation, whereas the mean consultation time each received from a urogynecologist was 1.7 h (0.3–12.5 h).

**Fig. 1:**
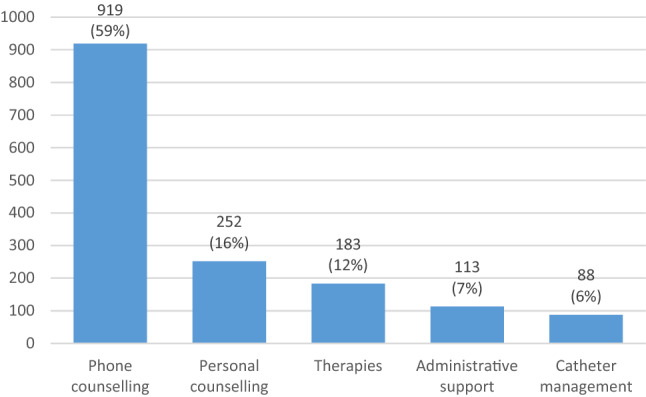
Activities of the urotherapist during the evaluation period (07/2016 to 06/2018)

Figure 1 provides an overview of the different urotherapist activities.

During the evaluation period, 132 (26%) of the 514 patients underwent surgery: 60, incontinence surgery (tension-free vaginal tapes, Botox and urethral bulking injections) and 72, pelvic organ prolapse surgery.

From the 1195 urogynecological patients who were not referred to the urotherapist, 280 (23%) directly underwent surgery: 140, incontinence surgery and 140, pelvic organ prolapse surgery.

### Subgroup collective

Patient demographics, diagnosis and consultation characteristics of the subgroup (38 patients) are listed in Table 1.The average patient age is significantly higher than the average age of the main collective (*p* = 0.038): 9 (24%) of the patients were < 65 and 29 (76%) were ≥ 65.

In all, the 38 patients of the study subgroup had 58 PFDs, i.e., there were 1.5 $\pm$ 0.7 PFDs per patient on average, which is significantly higher than in the main collective (*p* = 0.000):33 patients (87%) had an overactive bladder with or without incontinence/recurrent urinary tract infections/chronic cystitis/pelvic pain syndrome (summarized as increased bladder sensation complaints), 9 (24%) had pelvic organ prolapse, 10 had stress urinary incontinence (26%) and 6 (16%) had fecal incontinence.

The German Pelvic Floor Questionnaire, a self-administered questionnaire that integrates bladder, bowel and sexual function and pelvic organ prolapse, was used to record the symptoms of PFDs, their bothersomeness and the condition-specific quality of life [19]. The mean scores for the four domains, bladder, bowel, prolapse and sexual function, were 3.89 ($\pm$ 1.87) for the bladder domain, 2.66 ($\pm$ 1.61) for the bowel domain, 1.01 ($\pm$ 1.88) for the prolapse domain and 0.73 ($\pm$ 1.61) for the sexual function domain. The overall pelvic floor dysfunction score was 8.41 ($\pm$ 4.63).

The total number of urotherapeutic consultations of the 38 patients was 330: 17 patients (45%) had less than 5 consultations and 21 (55%) had 5 or more consultations. The types of consultation were as follows: 88 phone counsellings, 40 personal counsellings, 137 therapies (such as percutaneous posterior tibial nerve stimulation (PTNS) or pessary fitting), 56 catheter management appointments and 9 administrative support sessions.

In all, 17 of the 38 patients were ≥ 65 and had ≥ 5 consultations; 15 (65%) because of an overactive bladder with or without incontinence/recurrent urinary tract infections/chronic cystitis/pelvic pain syndrome (summarized as increased bladder sensation complaints).

On average, each patient in the study subgroup received 5.7 h (0.5–35.8 h) of urotherapist consultation, whereas the mean consultation time each received from a urogynecologist was 2.6 h (0.5–10.8 h).

From the total 215 h of urotherapist consultation, 189 h (88%) were personal contact with the patient and 27 h (12%) were contacts by phone or occasionally by email. A personal consultation took 47 min on average, and a consultation by phone took 27 min on average.

During the evaluation period, four patients underwent prolapse surgery and three patients had incontinence surgery: two tension-free vaginal tapes and one Botox injection in the detrusor vesicae.

### Patient satisfaction

Out of the study subgroup, 32 of the 38 patients (84%) returned the questionnaire on patient satisfaction. Table 2 summarizes the results and shows the high level of agreement with the professional competence, empathy, reachability, temporary availability, quality of advice and treatment success of the urotherapist and the urogynecologists.

**Table 2:** Patient satisfaction

	Strongly agree	Agree	Neither agree nor disagree	Disagree	Strongly disagree	No answer
Statement about the urotherapist						
Professional competence	26	6				
Empathy	25	7				
Reachability	20	10	2			
Temporary availability	18	14				
Quality of advice	26	6				
Treatment success	14	7	6	2	1	1
Statement about the urogynecologist						
Professional competence	27	4				1
Empathy	24	6	1			1
Reachability	19	8	3			2
Temporary availability	21	10				1
Quality of advice	24	7				1
Treatment success	13	7	8	2		2

	Will you take advantage of the urogynecological service/ of the urotherapist of our department again if necessary?	Will you recommend the urogynecological service/ the urotherapist of our department?
Yes	29 (91%)	29 (91%)
Maybe	1	1
No		
No answer	2	2

Self-administered questionnaire (Likert scale), *n* = 38, 32 returned

Both the reuse rate of the service provided by the urotherapist and the recommendation rate by the patients were high (91%).

The six patients (18%) who rated their treatment success with “neither agree nor disagree,” still specified their satisfaction with the service as “agree” or “strongly agree”. Two patients rated the success of their treatment as “disagree,” but were nevertheless satisfied with the service. One patient rated her treatment success as “strongly disagree” but would recommend the urotherapy to others.

## Discussion

The study took place in a tertiary referral hospital and a teaching hospital for gynecologists, urogynecologists and advanced nurse practitioners. One-third of the patients with PFDs were co-managed by the urotherapist. Age ≥ 65 and increased bladder sensation complaints proved to be the parameters pointing to the highest frequency of urotherapist consultations (Table1). The small study subgroup had a higher proportion of ≥ 65 patients, and thus, an even higher incidence of increased bladder sensation complaints (87%) was observed. Of the different lower urinary tract symptoms, increased bladder sensation complaints are the most time-consuming disorders overall [20–22]. The high inconvenience caused by increased bladder sensation complaints is also reflected in the scores of the German Pelvic Floor Questionnaire, which were highest in the bladder domain (Table 1). This corresponds to findings of a study by Agarwal et al. [22], where urinary urgency was the most common troubling symptom in a large population-based study.

Pelvic organ prolapse was the second most common PFD. The number of urotherapist consultations was lower for pelvic organ prolapse than for increased bladder sensation complaints (Table 1). It was previously reported that the counselling of patients with pelvic organ prolapse and the fitting of pessaries by an advanced nurse practitioner is an effective conservative treatment [23, 24]. Pessary treatment is gaining more importance as part of a urotherapy, especially since pelvic organ prolapse surgery is under debate due to recurrences and complication rates [25]. Pessaries are a safe and effective treatment option for women with pelvic organ prolapse; managing these patients needs person-centered care, which is a core activity of urotherapists [26].

The study revealed that most consultations took place by phone (59%). A small proportion of patients (16% of the main collective) needed five or more consultations, indicating that the comprehensive, competent management of some patients can be very time-consuming (Table 1). In chronic PFDs, urotherapy has a supportive, caring, long-term character and cannot be compared to treatment by medication or surgery.

The mean consultation time of the urotherapist per study subgroup patient was 5.7 h, which is more than twice the consultation time of the urogynecologist (Table 1). The length of the counselling time reflects the patient's need for information and education. In addition, the length of the consultation varies considerably; in general, older patients need more time as shown by our subgroup, which included significantly more patients ≥ 65 than the main collective.

Consultations by the theurotherapist varied widely from counselling for continence products or clarifying pre- and post-operative administrative issues to intense, long-term therapies, including percutaneous posterior tibial nerve stimulation (PTNS), bladder instillations or catheter management (inclusive instruction for self-catheterization). Patients with PFDs have different levels of personal requirements that have to be evaluated individually by the urotherapist. The questionnaire assessing patient satisfaction (Table 2) showed that this effort is highly appreciated by patients and, thus, likely to contribute to a high satisfaction with the service.

In multimorbid patients in need of tailored, person-centered care, among other nursing core components, urotherapy is deemed to be important in an effective integrated care model [27].

Evidence-based implementation theories should be applied when nurse-led integrated care models are developed and evaluated [28].

The reported study has limitations. First, because urotherapy was established as a new service, the data are only evaluated descriptively and were provided by one urotherapist, which makes the conclusions more difficult to generalize. Second, we did not employ a standardized written counselling protocol. Furthermore, we could not recruit as many patients for the subgroup as planned, which is not uncommon in real-life, observational studies [29]. Many patients found reading the ten pages of patient information too bothersome and declined participation in the study, but not management by the urotherapist. Another limitation is the high percentage of women (62%) that were satisfied with one or two urotherapist consultations and, subsequently, were no longer interested in participating in a substudy. When planning the study, it was impossible to predict how much time and effort the urotherapist would have to invest to adequately care for the patients according to their different PFDs; surprisingly, most patients needed only a limited number of consultations, mainly transmitted by phone.

Our study could not answer the question of whether or not the concept of an interprofessional approach has an impact on health costs [11]. In two recent reviews concerning interprofessional collaborations [7, 17] and in a Cochrane Database review from 2017 [9], the authors discuss how it is still not clear how the interprofessional collaboration concept should be realized. There is not sufficient evidence to draw conclusions on the effects of interprofessional interventions and whether or not this approach will have implications for a better and more economical health care system [7, 9, 17]. In the literature, greater efficiency and lower costs of an interprofessional approach are discussed controversially [30].

Despite the limitations, the study indicates that the care delivered by a urotherapist meets patients’ needs and can be extended to help treat the growing population of patients with PFDs. The integration of communication tools such as structured protocols for counseling by the urotherapist, specific hotline contact, telemedicine (for frail patients) and e-mail contact should be evaluated while taking into account issues regarding data safety (e.g., access to medical records). In addition, the results provide a good basis for negotiations with health insurances on covering patient-attributable costs.

By carrying out specialized activities that are related to the care of patients with chronic pelvic floor disorders, urotherapists could relieve the workload of urogynecologists, who would then have more time to deal with surgery and teaching. Further, as documented by the voluntary questionnaire (Table 2), nurse-led counselling covers a patient need in urogynecology.

In the future, the certification of an organization as a pelvic floor center could include the integration of a urotherapist as a prerequisite. Thus, the competencies and decision-making powers of the urotherapist in an interprofessional setting need to be precisely defined and further evaluated [31].

## Conclusions

The management of chronic diseases, such as PFDs, calls for different models in primary care compared to acute medicine where cure mostly occurs within a foreseeable time frame. We could demonstrate that partnership between a urotherapist and urogynecologists in an outpatient clinic is a key component in the effective management of PFDs in adult patients. Furthermore, the activities of the urotherapist were highly appreciated by the majority of the study subgroup patients and these patients would also recommend seeing a urotherapist to other women. The overview of urotherapist activities provided by this study will facilitate the further development of interprofessional collaborations and partnerships between advanced nurse practitioners and doctors. The inclusion of such collaborations in patient care plans has the potential to transform health care services for women with PFDs for the better.

## Electronic supplementary material

Below is the link to the electronic supplementary material.

Supplementary material 1 (DOCX 26 kb)

## Data Availability

The dataset used and analyzed for this submission is available from the corresponding author on reasonable request.
