# Supplementary material for: Urotherapist activities in caring for patients with pelvic floor disorders: a prospective single-center observational study
Source: Arch Gynecol Obstet. 2020 Sep 30;303(2):471–9. doi: 10.1007/s00404-020-05810-0 (PMC7858547; doi:10.1007/s00404-020-05810-0)
Supplement: Supplementary file 1 — Supplementary material 1 (DOCX 26 kb) [file 404_2020_5810_MOESM1_ESM.docx]

Appendix

**Questionnaire for Patient satisfaction**

Dear Patient,

After the treatment of your bladder and pelvic floor complaints, we would like to know your opinion about our service, in particular, the service you received from our urotherapist.

We seek to improve the quality of our treatments. By completing this questionnaire, you will make an important contribution for the benefit of future patients.

Thank you very much,

Your Urogynecology Team

**What is your statement about the care provided by the urotherapist**

|  | Strongly agree | Agree | Neither agree nor disagree | Disagree | Strongly disagree | No  answer |
| --- | --- | --- | --- | --- | --- | --- |
| Professional competence |  |  |  |  |  |  |
| Empathy |  |  |  |  |  |  |
| Reachability |  |  |  |  |  |  |
| Temporary availability |  |  |  |  |  |  |
| Quality of advice |  |  |  |  |  |  |
| Treatment success |  |  |  |  |  |  |

**What is your statement about care provided by the urogynecologist?**

|  | Strongly agree | Agree | Neither agree nor disagree | Disagree | Strongly disagree | No  answer |
| --- | --- | --- | --- | --- | --- | --- |
| Professional competence |  |  |  |  |  |  |
| Empathy |  |  |  |  |  |  |
| Reachability |  |  |  |  |  |  |
| Temporary availability |  |  |  |  |  |  |
| Quality of advice |  |  |  |  |  |  |
| Treatment success |  |  |  |  |  |  |

**Will you take advantage of the urogynecological service/ of the urotherapist of our department if necessary again?**

| Yes |  |
| --- | --- |
| Maybe |  |
| No |  |
| No answer |  |

**Will you recommend the urogynecological service/ the urotherapist of our department?**

| Yes |  |
| --- | --- |
| Maybe |  |
| No |  |
| No answer |  |

If you have any comments or questions, please formulate them here:
